# Retraction: Human gut microbiota: dysbiosis and manipulation

**DOI:** 10.3389/fcimb.2013.00104

**Published:** 2013-12-19

**Authors:** Frontiers in Microbiology Editorial Office

The authors and the journal wish to retract the 27 September 2012 article cited above. Based on information discovered after publication and reported to the journal in November 2013, this article was found to contain sections that were taken verbatim from other sources without giving proper reference to the source and without identifying the citation as a word-by-word citation from that source. The authors agree with retraction of the article and apologize to the readers, reviewers, and editors of *Frontiers in Cellular and Infection Microbiology*.

